# *Hox* gene expression during postlarval development of the polychaete *Alitta virens*

**DOI:** 10.1186/2041-9139-4-13

**Published:** 2013-05-01

**Authors:** Nadezhda I Bakalenko, Elena L Novikova, Alexander Y Nesterenko, Milana A Kulakova

**Affiliations:** 1 Department of Embryology, Laboratory of Experimental Embryology, SaintPetersburg State University, Oranienbaumskoe sh., 2. St. Peterhof, Saint Petersburg, Russia

**Keywords:** *Hox* genes, ncRNA, Polychaete, Positional information

## Abstract

**Background:**

*Hox* genes are the family of transcription factors that play a key role in the patterning of the anterior-posterior axis of all bilaterian animals. These genes display clustered organization and colinear expression. Expression boundaries of individual *Hox* genes usually correspond with morphological boundaries of the body. Previously, we studied *Hox* gene expression during larval development of the polychaete *Alitta virens* (formerly *Nereis virens*) and discovered that *Hox* genes are expressed in nereid larva according to the spatial colinearity principle. Adult *Alitta virens* consist of multiple morphologically similar segments, which are formed sequentially in the growth zone. Since the worm grows for most of its life, postlarval segments constantly change their position along the anterior-posterior axis.

**Results:**

We studied the expression dynamics of the *Hox* cluster during postlarval development of the nereid *Alitta virens* and found that 8 out of 11 *Hox* genes are transcribed as wide gene-specific gradients in the ventral nerve cord, ectoderm, and mesoderm. The expression domains constantly shift in accordance with the changing proportions of the growing worm, so expression domains of most *Hox* genes do not have stable anterior or/and posterior boundaries.

In the course of our study, we revealed long antisense RNA (asRNA) for some *Hox* genes. Expression patterns of two of these genes were analyzed using whole-mount *in-situ* hybridization. This is the first discovery of antisense RNA for *Hox* genes in Lophotrochozoa.

**Conclusion:**

*Hox* gene expression in juvenile *A. virens* differs significantly from *Hox* gene expression patterns both in *A. virens* larva and in other Bilateria.

We suppose that the postlarval function of the *Hox* genes in this polychaete is to establish and maintain positional coordinates in a constantly growing body, as opposed to creating morphological difference between segments.

## Background

The amazing diversity of body plans of bilateral animals is a result of the structural and regulatory evolution of genes directly connected to morphogenetic processes. Among these, *Hox* genes, which play a crucial role in regionalization of the anterior-posterior (AP) axis in bilateral animals, are particularly interesting [1,2]. These genes display clustered organization and colinear expression, and are highly conserved.

Current knowledge of the function of *Hox* genes, and even of their expression patterns, is nonuniform across different clades of Bilateria. While in Deuterostomia and Ecdysozoa *Hox* gene functions are well studied, at least for vertebrates and arthropods, research in Lophotrochozoa is still at the initial stage. There are only a few studies of this animal group that describe expression patterns of all *Hox* genes in the cluster [3-5]. However, the Lophotrochozoa group includes an unsurpassed amount of diverse body plans and is very promising for the study of mechanisms of morphogenetic evolution.

One of the major phyla among Lophotrochozoa is Annelida. Polychaeta is a basal class of Annelida [6]. Many species in this group have indirect development that includes stages of an unsegmented trochophore larva (a trait shared with many other Lophotrochozoa phyla) and a segmented larva, nectochaete. In the postlarval stage, polychaetes generate segments through a subterminal growth zone (GZ).

The diversity of polychaetes is manifest in various segment morphologies. There are species with morphologically similar segments, like representatives of the Nereididae family, as well as heteronomously segmented species that have different segments grouped into tagmata. To date, the expression of *Hox* genes has been studied in two heteronomously segmented polychaetes, *Chaetopterus* and *Capitella*. As in most bilateral animals, the expression boundaries of individual *Hox* genes in these species correspond with morphological boundaries of the body [5,7]. These expression patterns are consistent with the possible role of *Hox* genes in establishing morphological identity along the AP axis.

Our model object is a nereid polychaete *Alitta (Nereis) virens*. This is an errant homonomously segmented worm. The ontogenesis of this polychaete includes a lecithotrophic trochophore, a nectochaete with three seta-bearing segments and a multi-segmented worm that grows throughout most of its life. This body plan is likely to be basal among Polychaeta [8]. Previously, we completed a study of larval *Hox* gene expression in nereids *Alitta virens* and *Platynereis dumerilii*[4]. The genomes of these species contain the whole complement of *Hox* genes specific to Lophotrochozoa: *Hox1* (PG1), *Hox2*(PG2), *Hox3*(PG3), *Hox4*(PG4), *Hox5*(PG5), *Lox5*(PG6–8), *Hox7*(PG6–8), *Lox4*(PG6-8), *Lox2*(PG6-8), *Post2*(PG9+) and *Post1*(PG9+) [4]. The *Hox* genes in a segmented larva seem to define its body plan according to the principle of spatial colinearity, as they do in most bilaterian animals.

During postlarval development, *A. virens* continues to form new segments in the GZ for almost its whole life. *Alitta*’s body does not have any apparent morphological boundaries. This raises a question about the role of the *Hox* genes in such an animal.

Apart from morphological heteronomy of the segments, most polychaetes display primary segmental heteronomy, which is based on differences between larval and postlarval segments. This is characteristic of both heteronomously and homonomously segmented polychaetes. Many researchers point out significant dissimilarities in formation, structure and ability to regenerate larval and postlarval segments [9-11]. In particular, the larval segments form almost simultaneously by splitting the single somatic plate into metameres; they do not produce reproductive products, and do not have metanephridia (only protonephridia are present) [4,10,12]. Postlarval segments, on the other hand, are formed sequentially in the posterior GZ, have metanephridia, and can produce reproductive products. There are some groups of polychaetes that use only one type of segmentation. For example, Polygordiidae lack a segmented larva, and a juvenile worm is formed right after trochophore metamorphosis. Dinophilidae (Archiannelida), on the other hand, have only ‘larval’ segments. Sometimes within a single family (such as Nereididae), there are species that have all stages of development from trochophore to an adult segmented worm (*Platynereis dumerilii*), as well as species with direct development (*Neanthes arenaceodentata*) [10,13,14]. Finally, different individuals of the same species can have direct development as well as development through a larval stage; moreover, the larvae can be of different types (planktonic and benthic) [15,16].

The evolutionary and ontogenetic flexibility of polychaetes suggests that stages of their development are independent ‘modules’, controlled by different morphogenetic programs. In this case, we can expect that *Hox* gene expression patterns in larval and postlarval development are significantly different.

In this study, we describe detailed *Hox* gene expression patterns using whole-mount *in-situ* hybridization (WMISH) in polychaete *Alitta virens* during postlarval stages. We address three main questions. First, are *Hox* genes involved in patterning of postlarval segments? Second, are their expression patterns during postlarval development consistent with a conserved *Hox* gene function to convey morphological segment identity? Third, does *A. virens* have considerable differences between larval and postlarval *Hox* gene expression?

## Methods

### Animals

Adult *Alitta virens* were collected near the Kartesh Marine Biological Station of the Zoological Institute (RAS), at the White Sea, Chupa Inlet. Mature animals were caught with a hand net at the water surface during their spawning period (June and July). Artificial fertilization and cultivation of the embryos were carried out at 10.5°C [17]. A culture of postlarval animals was kept in the Laboratory of Experimental Embryology (Peterhof, Russia) under the following conditions: temperature −18°C, salinity −23‰, artificial seawater (Red Sea salt). The size of nechtochaetes is about 0.8 mm; 4 to 6 segment worms, 1 mm; 10 to 12 segment worms, 2 mm; 15 to 20 segment worms, 3 to 4 mm; worms with more than 20 segments, up to 6 mm.

### Cloning of *A. virens Hox* genes

The cloning of *A. virens Hox* genes was described previously [4,18,19]. Gene fragments for probe synthesis were received by 3'RACE. Gene fragments, except *Nvi-Hox3*, were inserted into pGEM®-T Easy Vector (Promega). *Nvi-Hox3* was inserted into pBluescript II SK^+^ (Fermentas). The vector sequence allows sense and antisense probes to be obtained from different promoters (T7 and Sp6). Riboprobes were generated from fragments of the following lengths: 548bp for *Nvi-Hox1*, 580bp for *Nvi-Hox2*, 550bp for *Nvi-Hox3*, 453bp for *Nvi-Hox4*, 1010bp for *Nvi-Hox5*, 573bp for *Nvi-Lox5*, 522bp for *Nvi-Hox7*, 302bp for *Nvi-Lox4*, 498bp for *Nvi-Lox2* and 380bp for *Nvi-Post2*.

### Whole-mount *in-situ* hybridization (WMISH)

Whole-mount *in-situ* hybridization was performed for *Alitta* as described previously [4] with the following modifications. Hybridization was carried out at 65°C, and washings from probes at 67°C. Collagenase treatment (collagenase (Sigma) 100 γ/ml, 2.5 mM dithiothreitol (DTT); 1 mM CaCl_2_) was performed for 5 to 10 min, proteinase K (Sigma) treatment was performed for 10 to 20 min (10 γ/ml). Washings from the probes were performed as follows: 100% Hybe 2 × 60 min, 80% prehybe/20% PTw 2 × 20 min, 50% prehybe/50% PTw 4 × 30 min, 20% prehybe/80% PTw 2 × 20 min, 100% PTw 2 × 20 min at 67°C. Washings from antibodies were carried out for 10 × 20 min in PTw on the shaker. The detailed protocol is available on request. Between 8 and 20 worms were used for each stage. BM-purple (Roche) was used as a chromogenic substrate to localize the hybridized probe. The time of incubation in substrate was 12 h for sense transcripts and 24 to 48 h for antisense transcripts and sense transcript of *Nvi-Lox4*. The worms were mounted in clove oil before microscopic analysis. We used the following negative control: animals taken through the entire *in-situ* protocol, but with no probe (see Additional file 1). The results were imaged on a DMRXA microscope (Leica) with a Leica DC500 digital camera with Nomarski optics. Optical sections were assembled with Helicon Focus software. Brightness, contrast, and color values were corrected in all images using Adobe Photoshop CS5 image processing software.

## Results

### Brief description of *Alitta virens* postlarval development

**Figure 1:**
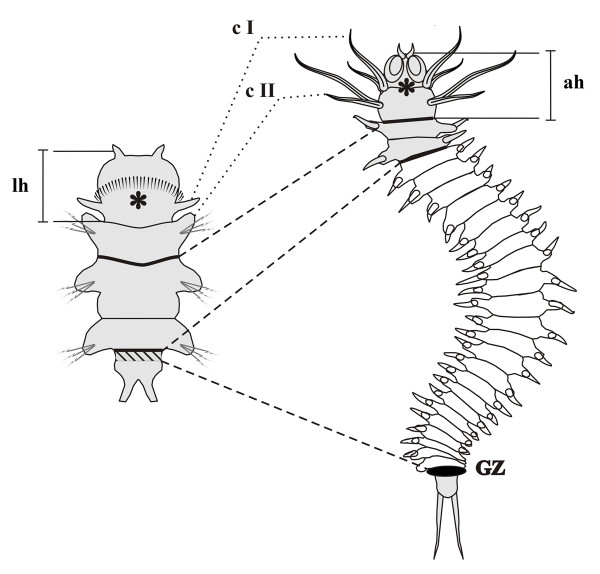
**General scheme matching larval (left side) and adult (right side) body structures of Alitta virens.** IHC staining for β-catenin and Nanog expression in NSCLC. Representative images of intracellular β-catenin expressed at the membrane (**A**), in cytoplasma (**B**) or in the nucleus (**C** and **D**). In serial sections, Nanog staining (**E** and **F**) frequently overlapped with β-catenin staining (**C** and **D**) in the nuclei of most cells.

**Figure 2:**
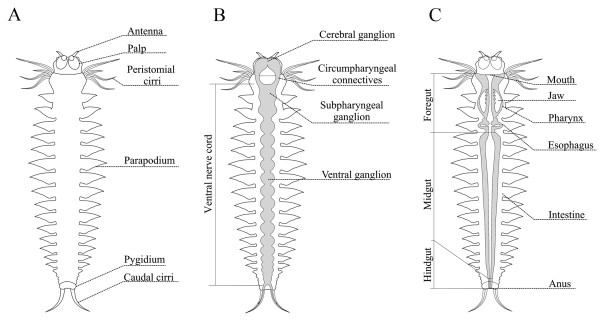
**Anatomy of Alitta virens.** (**A**) General view. (**B**) Structures of central nervous system. (**C**) Structures of digestive system.

The main stages of *Alitta virens* larval development were described previously [4]. The postlarval growth begins with origination of the fourth segment anlage (this is the first postlarval segment) from posterior GZ (Figure 1). Postlarval segments form sequentially, one by one, in the GZ. When a worm has 6 to 8 segments, its first chaete-bearing segment (the first larval segment) changes significantly. Its parapodia are considerably reduced, they lose their chaetae, their cirri grow and transform into a second pair of peristomial cirri. Then, the first larval segment becomes part of the peristome of the adult head. After this stage, the second larval segment becomes the first body segment of a juvenile worm (Figure 1). Formation of new segments continues for most of *Alitta*’s life and stops when the worm has about 200 segments. The morphology of the juvenile worm is shown in Figure 2.

### *Nvi-Hox* gene expression patterns

To bring descriptive data for expression patterns into a system, we divided ten *Hox* genes into groups based on their expression behavior during larval development [4]. As in larval development [20], *Nvi-Post1* is not detectable in broad ectodermal domains of juvenile worms. *Nvi-Post1* expression was observed in the chaetal sacs of developing chaetae (data not shown). Apparently, its expression is not related to the AP patterning, and we will not discuss it here. In general, most *Nvi-Hox* genes are expressed in parapodia; this calls for a separate study and is not covered in this article.

### *Nvi-Hox1*, *Nvi-Hox4*, *Nvi-Hox5*, *Nvi-Lox5*, *Nvi-Post2*

The first group includes *Nvi-Hox1*, *Nvi-Hox4*, *Nvi-Hox5*, *Nvi-Lox5*,and *Nvi-Post2*. These genes have colinear expression patterns during larval development.

### *Nvi-Hox1*

**Figure 3:**
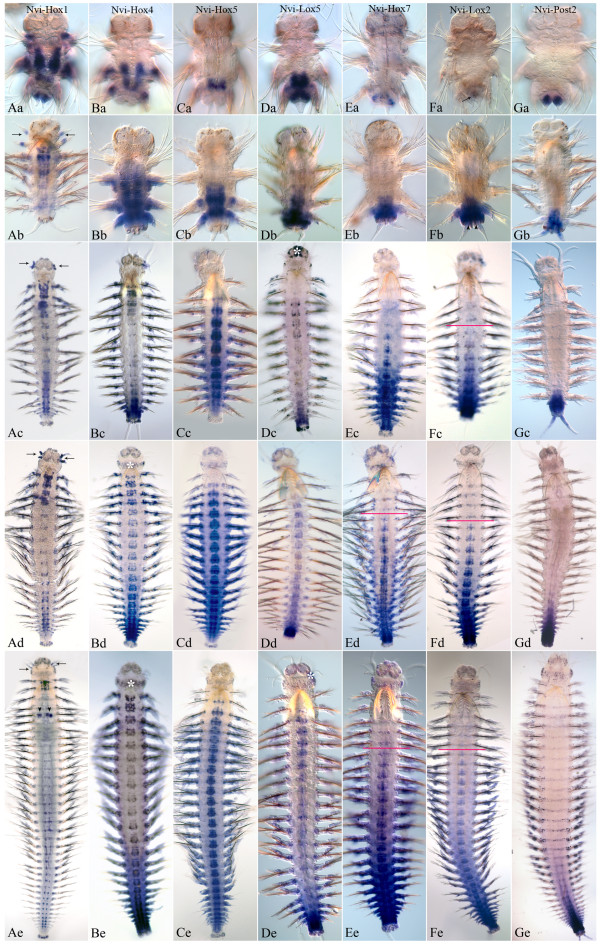
**Expression of Nvi-Hox genes in nectochaetes and juvenile worms.** Nvi-Hox gene expression patterns at the nectochaete stage are shown in the top row. Juvenile worms before adult head formation are in the second row. In the third, fourth, and fifth rows there are juvenile worms with 10 to 12, 15 to 20, and more than 20 segments, respectively. The anterior end is directed upwards on all panels. All views are ventral. White asterisks mark the background. Expression patterns of Nvi-Hox1 are shown in panels (**Aa–Ae**). Arrows show expression in peristomial cirri. Arrowheads on the panel Ae indicate Nvi-Hox1 expression in the pharynx. (**Ba–Be**) Expression of Nvi-Hox4. (**Ca–Ce**) Expression of Nvi-Hox5. (**Da–De**) Expression of Nvi-Lox5. (**Ea–Ee**) Expression of Nvi-Hox7. Red lines indicate the anterior border of Nvi-Hox7 expression in the VNC. Metameric staining in several anterior segments is a background in epithelial glands. These are pigmented and tend to bind antibodies nonspecifically (shown in higher magnification in Figure 6F). (**Fa–Fe**) Expression of Nvi-Lox2. The arrow shows weak expression at the nectochaete stage. Arrowheads in panel Fb point the Nvi-Lox2 expression in the pygidium area. Red lines indicate the anterior border of Nvi-Lox2 expression in the VNC. Metameric staining in several anterior segments is a background in epithelial glands. (**Ga–Ge**) Expression of Nvi-Post2. See text for details.

At the end of the larval development at the late nectochaete stage, expression domain of this gene has a sharp boundary in the first larval segment (Figure 3Aa). *Nvi-Hox1* is detected in the ganglia of the ventral nerve cord (VNC) and parapodial ectoderm of all three larval segments with the highest level in the second segment and the lowest level in the third segment. In addition, *Nvi-Hox1* expression marks the bases of peristomial and pygidial cirri. After metamorphosis, the expression expands to include the fourth and the fifth segments (first and second postlarval segments) (Figure 3Ab). Expression in pygidial cirri is significantly reduced. In juvenile worms with six to ten segments, two new *Nvi-Hox1* expression domains appear: in the digestive tube at the foregut-midgut boundary (Additional file 2) and in the lateral part of the ganglia of the new segments. The expression patterns in anterior and posterior ganglia are different. In anterior segments, expression is detected in most of the ganglia but in posterior segments, *Nvi-Hox1* expression appears as two spots in the lateral part of each ganglion (Figure 3Ac-Ae, Additional file 2). Despite significant changes in the first segment associated with the reorganization of the peristome, the anterior border of *Nvi-Hox1* expression in this segment is persistent (Figure 3Ab-Ae; Figure 3A,E). *Nvi-Hox1* expression during later postlarval stages forms a gradient with two maxima: in the first body segment (second larval segment) and in the ganglia of posterior third of the body. *Nvi-Hox1* expression in the medial segments is weak. The most posterior and youngest segments and the GZ are *Nvi-Hox1*-negative (Figure 3Ab-Ae).

### *Nvi-Hox4*

During larval development, *Nvi-Hox4* expression is associated with development of the second larval segment. At the nectochaete stage, expression extends to include the third segment, but is not detected in the pygidium (Figure 3Ba).

**Figure 4:**
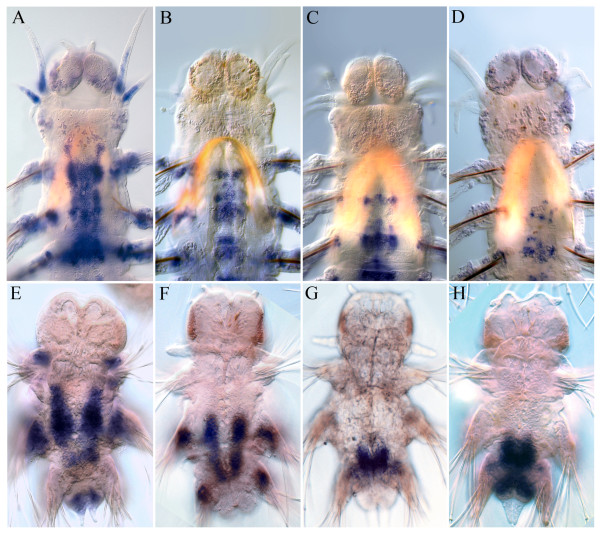
**Anterior boundaries of *Nvi-Hox1 *(A, E), *Nvi-Hox4 *(B, F), *Nvi-Hox5 *(C, G), and *Nvi-Lox5 *(D, H) have been stable since the larval stages.** Anterior ends of juvenile worms are in the top row, nectochaetes are in the bottom row. See text for details.

At the onset of postlarval growth, strong *Nvi-Hox4* expression is detectable in the ganglia and ectoderm of each nascent segment (Figure 3Bb). In small worms (6 or 7 segments), *Nvi-Hox4* expression takes on a posterior-anterior gradient (data not shown). The interior border of *Nvi-Hox4* expression is retained in the second larval segment. After the first larval segment has merged with the peristome, the second larval segment becomes the first adult body segment. *Nvi-Hox4* expression persists in this area during all postlarval stages (Figure 3Bb-Be). In animals larger than ten segments, the VNC expression gradient changes its shape. Now *Nvi-Hox4* is expressed intensively in several anterior ganglia and in the most posterior ones, but staining is much weaker in central segments. In the ectoderm *Nvi-Hox4* expression is strong in the most posterior newly formed segments and decreases gradually toward the anterior end so it is no longer detectable in the middle part of the body. Both gradients (in the VNC and the ectoderm) ‘stretch’ but keep their shapes at all analyzed stages (Figure 3Bc-Be). The anterior border of *Nvi-Hox4* expression in VNC is stable and located in the first body segment (Figure 3Bb-Be, Figure 4B,F).

### *Nvi-Hox5*

During larval development, expression of this gene begins later than *Nvi-Hox1* or *Nvi-Hox4*[4] and is limited to the neuroectoderm of the third segment (Figure 3Ca). Since the beginning of postlarval growth, the *Nvi-Hox5* domain expands to include the ectoderm of this segment and the fourth segment surface and the developing neuromere (Figure 3Cb). In juvenile *Alitta virens*, *Nvi-Hox5* is expressed in each postlarval segment that already has parapodia. Several youngest, most posterior segments are *Nvi-Hox5*-negative. Moreover, *Nvi-Hox5* expression is not detectable in the pygidium and the GZ at all analyzed stages (Figure 3Cb-Ce). The expression pattern of this gene takes on a broad bow-shaped gradient. The anterior border of *Nvi-Hox5* expression is retained in the second body segment (third larval segment) (Figure 3Cc-Ce; Figure 4C,G). The anterior boundary of the expression gradient in the ectoderm is located two or three segments posterior to that of the VNC. The *Nvi-Hox5* expression pattern does not change significantly during postlarval growth (Figure 3Cc-Ce).

### *Nvi-Lox5*

This gene displays early expression in larva during the third segment development [4]. At the late larval stages, expression persists in the third segment and expands to include the posterior GZ (Figure 3Da). After metamorphosis, the nascent postlarval segment displays strong *Nvi-Lox5* expression, which gradually decreases toward the anterior end. Staining in the third larval segment is slightly downregulated but does not disappear (Figure 3Db). In worms larger than ten segments, *Nvi-Lox5* forms the expression gradient in VNC similar to *Nvi-Hox4*. *Nvi-Lox5* expression has two peaks: one in several anterior ganglia and one in posterior segments. The transcription level of *Nvi-Lox5* is weak in the central part of *Alitta*’s body (Figure 3Dc-De). Expression in the second body segment is weak but persists at all analyzed postlarval stages (Figure 3Db-De; Figure 4 E,H).

### *Nvi-Post2*

At the nectochaete stage, *Nvi-Post2* expression is restricted to the pygidium and anal cirri (Figure 3Ga). At the onset of postlarval segmentation, expression of this gene expands to the GZ and new postlarval segments (Figure 3Gb). As the segment moves toward the anterior end, *Nvi-Post2* expression weakens and gradually disappears (Figure 3Gb-Ge). *Nvi-Post2* displays a short posterior-to-anterior expression gradient in the VNC, segmental ectoderm, mesoderm, and distal gut. The number of *Nvi-Post2*-positive segments increases with the growth of the worm, but their percentage of the total number remains virtually the same (Figure 3Gb-Ge).

### *Nvi-Hox7*, *Nvi-Lox4*, *Nvi-Lox2*

Expression of these genes starts at the later stages of larval development. Their expression zones overlap in prospective posterior GZ [4]. They do not take part in larval morphogenesis.

### *Nvi-Hox7*

In the late nectochaete and four-segment juvenile worm, *Nvi-Hox7* expression is restricted to the posterior GZ (Figure 3Ea). Expression is initiated in postlarval segments after the formation of the fourth segment (Figure 3Eb). In older worms, the*Nvi-Hox7* expression pattern takes on a posterior-to-anterior gradient in the VNC, segment ectoderm, and parapodia (Figure 3Ec-Ee). The expression gradient is proportional to the length of the growing worm body. (Figure 3 Ec-Ee). The anterior expression boundary is in the third segment (Figure 3Eb) in four-segment worms, and moves backwards as the worm continues to grow. In animals with 15to 30 segments, it is located in the fifth to seventh segment (Figure 3Ec-Ee), but since the staining weakens towards the anterior end, we cannot determine the exact position of the anterior boundary.

### *Nvi-Lox4*

**Figure 5:**
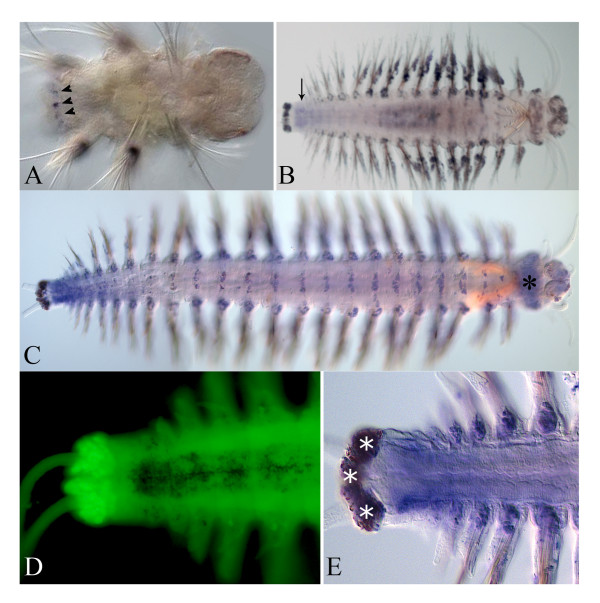
***Nvi-Lox4 *expression pattern during *Alitta virens *development.** The anterior end is to the right on all panels. All views are ventral. Asterisks mark a background. (**A**) Expression of *Nvi-Lox4* at the nectochaete stage is weak (marked with arrowheads). (**B**, **C**) Expression of *Nvi-Lox4* in juvenile worms (marked with arrow on panel **B**). On panel **C,** the metameric spots in each segment are a background in the epithelial glands. (**D**, **E**) Posterior part of the juvenile worm. (**D**) The worm was stained with To-Pro-1 (515/531). Expression of *Nvi-Lox4* is noticeable only in the VNC surface cells, as internal expression domains are hidden by fluorescence. There is no expression in the ectodermal part of the GZ. (**E**) Posterior part of the juvenile worm, deep view. *Nvi-Lox4* is expressed in the mesoderm. See text for details. GZ, growth zone; VNC, ventral nerve cord.

At all stages, detected *Nvi-Lox4* expression is very weak, which may be because of the small size of the RNA probe (302 b). *Nvi-Lox4* is first detected at the late nectochaete stage in a small number of cells in the anterior part of the pygidium (Figure 5A). In juvenile worms, the expression level increases with age. The *Nvi-Lox4* transcript is spread diffusely in VNC and does not display a sharp metameric pattern (Figure 5B,C). Expression is not detected in the ectoderm within the GZ and in the pygidium (Figure 5D). Low-level expression is detected in the mesoderm of the nascent segments (Figure 5E). *Nvi-Lox4* displays a posterior-to-anterior expression gradient (Figure 5C,D). The anterior border of *Nvi-Lox4* expression is loose and apparently not stable, like *Nvi-Hox7*.

### *Nvi-Lox2*

This expression is very weak in the nectochaete (Figure 3Fa), but intensifies significantly with the onset of postlarval segmentation (Figure 3Fb-Fe). The *Nvi-Lox2* transcript is detected in the posterior GZ, neural ganglia, and ectoderm of posterior postlarval segments and in the pygidium (Figure 3Fb). *Nvi-Lox2* also displays a posterior-to-anterior expression gradient, like that for *Nvi-Hox7*, *Nvi-Lox4*, and *Nvi-Post2*. Expression in the VNC lasts longer than in the segment ectoderm (Figure 3Fc-Fe). The anterior border of the *Nvi-Lox2* expression domain is unstable and constantly shifts toward the posterior end. The anterior expression boundary is in the third segment (Figure 3Fb) in four-segment worms, and moves backwards as the worm continues to grow. In animals with 15 to 30 segments, it is located in the sixth to eighth segment (Figure 3Fc-Fe), but since the staining weakens towards the anterior end, we cannot determine the exact position of the anterior boundary.

### *Nvi-Hox2*, *Nvi-Hox3*

These genes are expressed in an intensive and dynamic manner during early larval development [4], but at the nectochaete stage their expression domains narrow considerably.

### *Nvi-Hox2*

**Figure 6:**
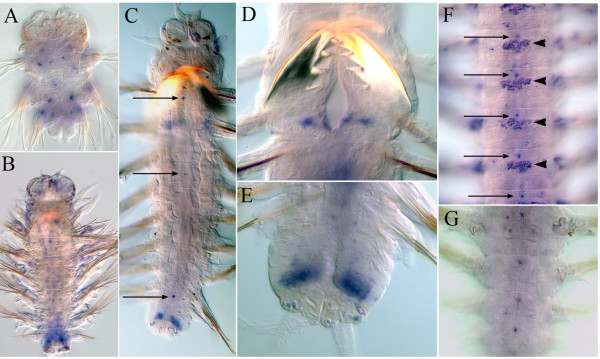
**Expression of *Nvi-Hox2 *in nectochaete (A) and juvenile worms (B-G).** The anterior end is directed upwards on panel **A**, and to the right on panels **B-G**. (**A**) *Nvi-Hox2* expression at the nectochaete stage. (**B**) *Nvi-Hox2* expression in small juvenile worm. (**C**) *Nvi-Hox2* expression in juvenile worm, general view. Arrows point to the expression domains in small patches of 3 to 5 cells at the midline. (**D**) *Nvi-Hox2* expression in the pharynx. (**E**) Posterior part of juvenile worm, deep view. *Nvi-Hox2* is expressed in the deep layer of GZ and the posterior segment. (**F**, **G**) Expression domains on the midline. (**F**) Several medial body segments. Expression of *Nvi-Hox2* is detected in small domains in each segment. Arrows point to the expression domains. Arrowheads mark a background in epithelial glands. (**G**) Several posterior segments. Expression of *Nvi-Hox2* is detected in small domains in each segment. See text for details. GZ, growth zone.

In the nectochaete, *Nvi-Hox2* is detected in small patches of cells in all larval segments (Figure 6A). A new expression domain appears in the mesodermal part of the GZ after metamorphosis (Figure 6B,C,E). Weak diffuse expression of *Nvi-Hox2* is detected in nascent segments in small juvenile worms (Figure 6B). Older worms do not have this expression domain. At the late juvenile stages, *Nvi-Hox2* is expressed in the mesoderm of the GZ and in small patches of 3 to 5 cells at the midline within each segment (Figure 6C,F,G). In worms of 8 to 10 segments and larger worms, *Nvi-Hox2* is also detected in the pharynx (Figure 6E).

### *Nvi-Hox3*

**Figure 7:**
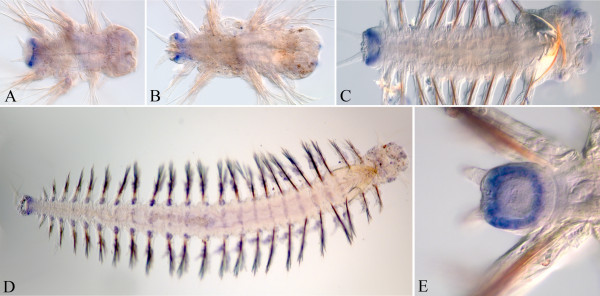
***Nvi-Hox3 *expression pattern in nectochaete (A) and at different stages of postlarval development (B-D).** The anterior end is to the right on all panels. (**B**) 4-segment worm. (**C**) 10-segment worm. (**D**) 25-segment worm. (**E**) Pygidium at higher magnification. See text for details.

Since the early nectochaete stage, *Nvi-Hox3* expression is present in the GZ. At all analyzed juvenile stages, strong, ring-like expression is detected in ectodermal cells of the GZ (Figure 7A-E).

### Antisense transcripts

We found antisense transcripts for some *Nvi-Hox* genes (*Nvi-Hox1, Nvi-Hox4, Nvi-Hox5*, *Nvi-Hox7*). Expression of *Nvi-antiHox5* and *Nvi-antiHox7* was analyzed in detail using WMISH.

### *Nvi-antiHox5*

**Figure 8:**
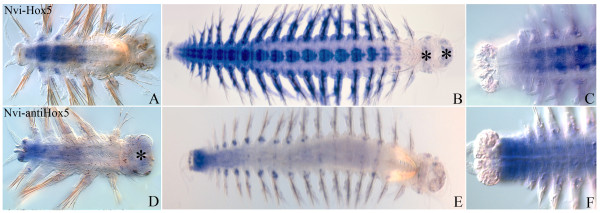
**Expression of *Nvi-Hox5 *(top row, panels A, B,C) and *Nvi-antiHox5 *(bottom row D, E, F).** The anterior end is to the right on all panels. All views are ventral. Asterisks mark a background. (**A, D**) Expression in small worms (5 or 6 segments). (**B, E**) Expression in juvenile worms (overview). (**C, F**) Posterior ends at higher magnification are shown. Expression domains of *Nvi-Hox5* (**C**) and *Nvi-antiHox5* (**F**) are complementary. See text for details.

After the beginning of postlarval segmentation, *Nvi-antiHox5* is detectable in the GZ and the posterior segments. Sense and antisense transcripts have adjacent and even slightly overlapping expression domains. The *Nvi-antiHox5* expression domain is more posterior than sense *Hox5* (Figure 8A,D). In a juvenile worm, *antiHox5* is detected in the GZ and the most posterior segments, where sense *Nvi-Hox5* is absent (Figure 8B,C,E,F). During postlarval development, *Nvi-antiHox5* expression does not have a stable anterior border. Its expression domain constantly shifts toward the posterior end.

### *Nvi-antiHox7*

**Figure 9:**
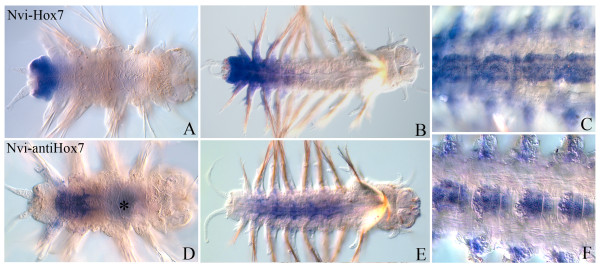
**Expression of *Nvi-Hox7 *(top row, panels A, B and C) and *Nvi-antiHox7 *(bottom row, panels D, E and F).** The anterior end is to the right on all panels. All views are ventral. Black and white asterisks mark the background. (**A, D**) Expression in 4-segment worm. (**B, E**) Expression in juvenile worms of about 10 segments. (**C, F**). Several central segments of the juvenile worms of about 20 segments at higher magnification are shown. Expression patterns for *Nvi-Hox7* (**C**) and *Nvi-antiHox7* in VNC are different. See text for details.

After the beginning of postlarval growth, this transcript is detected in VNC of the third and fourth segments anterior in relation to the sense transcript (Figure 9A,D). The anterior border of *Nvi-antiHox7* expression is always closer to the head than the sense transcript. Unlike the *Nvi-Hox7* sense transcript, *Nvi-antiHox7* is absent from nascent segments and the GZ (Figure 9B,E). In older worms, sense and antisense *Nvi-Hox7* domains seem to be broadly overlapping, but particular expression patterns in the same ganglia seem to be different (Figure 9C,F). These RNAs are probably expressed in different neurons.

## Discussion

### Summary of *Hox* gene expression

**Figure 10:**
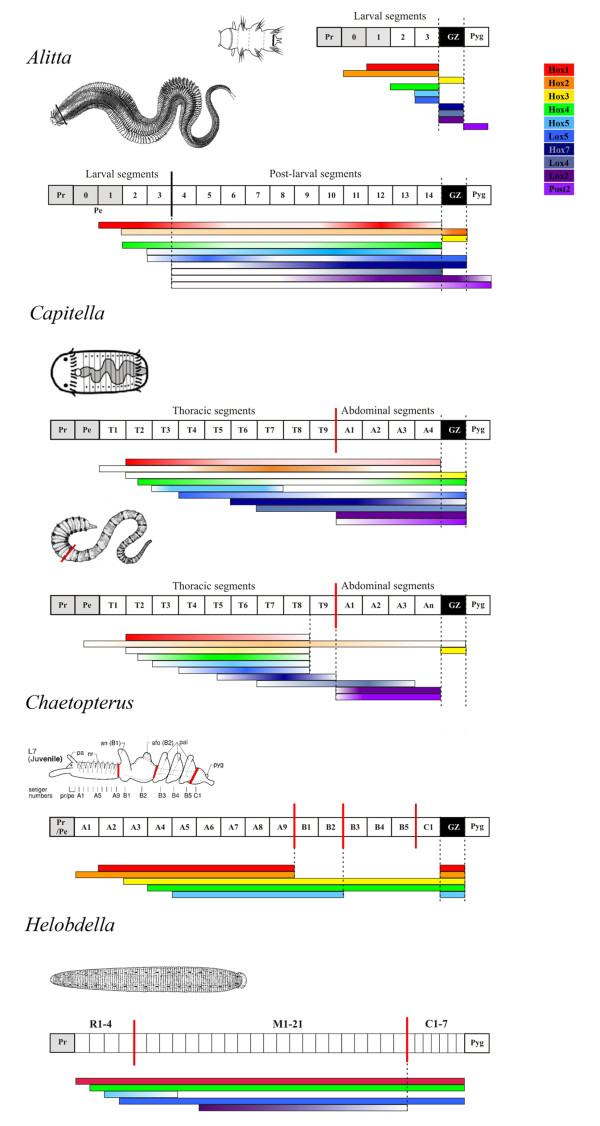
**Comparison of *Hox *gene expression patterns across annelids.** Generalized diagram comparing *Hox* gene expression along the main body axis of *Alitta virens* with the *Hox* expression in *Capitella*, *Chaetopterus*, and *Helobdella*. Body axes are shown as boxed diagrams next to the species names and include segments and tagmata for each species. Black vertical lines mark the boundary between larval and postlarval segments for *Alitta virens*, red vertical lines mark tagmata borders for other species. For *Alitta* and *Capitella*, expression is shown at late larval stages and for juvenile animals. Solid color bars indicate uniform expression level, while gradient color bars indicate gradient expression. Larval *Nvi-Hox* gene expression patterns have stable colinear expression boundaries like other polychaetes *Hox* genes. During postlarval development, most *Nvi-Hox* genes have gradient expression patterns. During worm growth, anterior boundaries of *Nvi-Hox7*, *Nvi-Lox4*, *Nvi-Lox2*, and *Nvi-Post2* shift from the fourth (first postlarval) segment toward the posterior end, while the posterior boundaries of *Nvi-Hox1* and *Nvi-Hox5* move toward the anterior end. *CapI-Hox* genes have stable expression boundaries corresponding to the morphological tagmata boundaries, but expression of many *Hox* genes is not uniform and forms a gradient. Abbreviations: GZ, growth zone; Pe, peristome; Pr, prostomium. Taxon-specific abbreviations: *Capitella*: A, abdominal segments; T, thoracic segments; *Chaetopterus*: A, B, C, segments of the body regions A, B, and C, respectively; *Helobdella*: C, caudal segments; M, medial segments; R, rostral segments. See text for details.

According to our results, most *Nvi-Hox* genes (*Nvi-Hox1*, *Nvi-Hox4*, *Nvi-Hox5*, *Nvi-Lox5*, *Nvi-Hox7*, *Nvi-Lox4*, *Nvi-Lox2*, and *Nvi-Post2*) form expression gradients in the central nervous system and ectoderm of *A. virens*. These gradients overlap significantly, but their span and shape are unique for each gene (Figures 3,5,10).

We do not have any data on physical linkage of *Alitta Hox* genes. However, among Lophotrochozoa, the cluster organization of *Hox* genes was shown for *Capitella sp. I*[5]. We presume that the genomic order of the *Hox* genes of *A. virens* is similar to that of *Capitella sp. I*.

The spatial colinearity is clearly present for *Nvi-Hox1*, *Nvi-Hox2*, *Nvi-Hox4*, *Nvi-Hox5*,and *Nvi-Lox5* genes, which have well-defined anterior boundaries (Figures 4). For *Nvi-Hox1*, *Nvi-Hox4*, *Nvi-Hox5*,and *Nvi-Lox5*, these boundaries were established during larval development (Figure 4). Anterior boundaries of other *Hox* genes (*Nvi-Hox7*, *Nvi-Lox4*, *Nvi-Lox2*, and *Nvi-Post2*) are not so clear, but the spatial organization of their expression domains also shows some colinearity. The *Nvi-Hox7* expression gradient spreads further towards the anterior end of the body than the gradients of *Nvi-Lox2* and *Nvi-Post2*. *Nvi-Post2* displays the shortest posterior-to-anterior gradient (Figure 3Ec-Ee,Fc-Fe,Gc-Ge). According to our results, the *Nvi-Lox4* anterior boundary lies posterior to the anterior boundary of *Nvi-Lox2* expression. This seems to violate the spatial colinearity principle. However, the detected expression of *Nvi-Lox4* is very weak; therefore, we cannot ascertain the true localization of its anterior boundary. The only gene that does not display any colinear expression is *Nvi-Hox3*, which is expressed in the GZ of the worm from the nectochaete stage.

### *Hox* gene expression in annelids

In most bilateral animals, *Hox* genes regionalize the AP axis of the body. A discrete distribution of Hox proteins divides the early embryo into separate domains by differential regulation of target genes. This leads to establishment of expression domains correlated with morphological boundaries of the body regions [21-23]. *Hox* gene expression in previously studied annelids is consistent with this general principle [5]. At the moment there are only a few studies concerning expression of *Hox* genes in annelids. Among these are studies on the expression of some *Hox* genes in larval development of *Chaetopterus sp.*[7] and *Platynereis dumerilii*[4], development of the leech *Helobdella sp.*[24], and larval and postlarval development of the polychaete *Capitella sp.I*[5].

Among all studied polychaetes, *Chaetopterus* has the most morphologically complex larva. It consists of three tagmata (A, B, and C), and there are morphological differences not only between segments in different tagmata, but also within a single tagma (В). At the late larval stages, posterior boundaries of *СН-Нох1* and *СН-Нох2* expression coincide with the boundary between tagmata A and B, and posterior boundary of *СН-Нох5* expression coincides with the border between morphologically diverse segments within tagma B (Figure 10) [7].

The *Capitella sp. I* body consists of a thoracic region, which includes nine larval segments, and an abdominal region, which includes four additional larval and all of the postlarval segments. There is no great morphological difference between thoracic and abdominal segments. During development, expression boundaries of some *Hox* genes are stabilized in the region between thoracic and abdominal tagmata (Figure 10) [5].

Even leeches, being highly specialized annelids, retain the axial specification of the nervous system by means of the *Hox* cluster. The leech *Helobdella sp.* consists of 32 segments: 4 anterior R1-R4, 21 central М1-М21, and 7 caudal С1-С7. The body nervous system of the leech is patterned by *Hox* genes according to the principle of spatial colinearity. Anterior expression boundaries of *He-Hox7* (*Hox1*), *He-Lox6* (*Hox4*), *He-Lox20* (*Hox5*),and *He-Lox5* genes correspond to nervous system structures in four sequential segments of the rostral region, while the posterior boundary of *He-Lox2* expression correlates with the anterior border of the caudal ganglion (Figure 10) [24].

*A. virens* and *P. dumerilii* have larvae with morphologically similar segments. The anterior expression boundaries of *Hox* genes coincide with the segments’ borders (*Nvi-Hox1*, *Nvi-Hox2*, *Nvi-Hox4*, *Nvi-Hox5*, *Nvi-Lox5*). The posterior boundaries are located between the segmented area and the pygidium. The *Nvi-Post2* gene marks the pygidium territory (Figure 10) [4].

During most of its postlarval life, *A. virens* continues to form morphologically identical segments, which are not divided into tagmata. In this case, most *Hox* gene expression domains do not possess the stable posterior boundaries that lie in the nascent segments or in the GZ. The expression domains of *A. virens Hox* genes cover most of the body and overlap significantly. Comparison of the *Hox* gene expression in annelids with different body plans confirms that the presence of posterior expression boundaries correlates with the presence of different body tagmata, as previously shown for other bilaterian animals, for example, Arthropoda [22].

### Larval and postlarval developmental programs

As mentioned, there are significant differences in formation of larval and postlarval segments of the polychaetes. These differences indicate the primary heteronomy of polychaete segments. Our data on *Hox* gene expression during ontogenesis of *A. virens* reveals the differences in molecular mechanisms of patterning of larval and postlarval segments.

**Figure 11:**
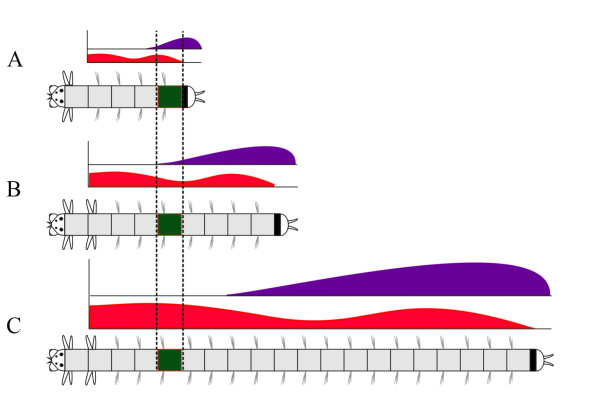
**Changes in *Hox *gene expression profile in the postlarval segment during worm growth.** As an example, the expression of two *Hox* genes is shown in one particular segment (highlighted in green). The expression gradient with two maxima of *Nvi-Hox4* is shown in red. The posterior-to-anterior gradient of *Nvi-Post2* is shown in purple. (**A**) The nascent postlarval segment of the small worm is the most posterior. In this case, both *Nvi-Post2* and *Nvi-Hox4* are strongly expressed in this segment. (**B**) As the worm grows and new segments are added, the highlighted segment moves to the central part of the body. Now, both *Nvi-Post2* and *Nvi-Hox4* expression is weak (for *Nvi-Hox4* this is a local minimum of expression level). (**C**) At the later stage, the highlighted segment is in the anterior part of the body. *Nvi-Post2* is no longer expressed in this segment, while for *Nvi-Hox4* this position corresponds to the anterior expression maximum.

First, a certain subset of *Hox* genes is expressed in each larval segment. Meanwhile, the postlarval segments express all *Hox* genes but not at the same time during postlarval growth. Each nascent segment expresses all *Hox* genes except *Nvi-Hox1*, *Nvi-Hox3*, and *Nvi-Hox5*. As new segments continue to form, the older segments move towards the head and begin to express *Nvi-Hox1* and *Nvi-Hox5*, while the expression of other genes is gradually downregulated or upregulated according to the shape of gene expression gradients (Figure 11). Interestingly, neural expression of *ParaHox* cluster genes in *A. virens* is governed by the same principle; each *ParaHox* gene is expressed at different periodsin all postlarval (but not the larval) neuromeres [25].

Second, three *Hox* genes, *Nvi-Hox7*, *Nvi-Lox4* and *Nvi-Lox2*, are not expressed during larval segment formation [4]. They are switched on in each postlarval segment on its formation in the GZ (*Nvi-Hox7*, *Nvi-Lox2*) or at the beginning of growth (*Nvi-Lox4*).

Taken together, the existing morphological data and the differential character of *Hox* gene expression in larval and postlarval development of *A. virens* support the idea of separate morphogenetic developmental programs in the segmented larva and adult worm.

Comparing *Hox* gene expression in different polychaetes (*Chaetopterus*, *Capitella*, *Platynereis*, *Alitta*), one can notice the fundamental similarity of *Hox* gene expression in their larval development. In all four cases (Figure 10), *Hox* genes are activated early in wide spatial domains of the larval body. The *Hox* gene expression corresponds to the location of primordial structures organized along the main body axis, and the expression anterior boundaries are colinear. These features link the program of polychaete larval body organization to the programs utilizing the *Hox* genes during the embryonic development of animals in other evolutionary clades, such as Deuterostomia and Ecdysozoa.

Apart from *A. virens*, *Hox* gene expression in only one other postlarval polychaete has been studied to date, *Capitella sp. I*[5]. Unfortunately, it is difficult to compare these two worms. All the segments of *A. virens* formed from the subterminal GZ are considered to be postlarval. In contrast, several segments of *Capitella* that are referred to as larval ones are formed sequentially from the posterior GZ [26]. The *Hox* gene expression in *Capitella* after metamorphosis is considerably different from that in *Alitta*. First, in postlarval segments only *CapI-Lox4*, *CapI-Lox2*, and *CapI-Post2* genes are active. Second, *Hox* gene expression in a juvenile worm is maintained only within the neural system ganglia. Third, expression boundaries in the juvenile worm are stable and correspond to the morphological tagma boundaries.

Nevertheless, a comparison of *Hox* ortholog expression in late *Capitella* larva, when it has already formed several segments from the posterior GZ, and juvenile *A. virens* reveals fundamental similarities in many gene patterns. *Hox1*, *Hox4*, and *Lox5* genes have anterior as well as posterior expression domains in both species (Figure 11). In both cases, the expression has a gradient shape (Figure 3) [5]. We can speculate that this stage in *Capitella* development is a separate phase that can be compared to the postlarval development of *A.virens*.

There are also significant differences of *Hox* gene expression between larval and juvenile stages of *Capitella* development. In juvenile stages of *Capitella*, almost all expression domains of *Hox* genes become limited to the VNC and discrete posterior expression boundaries appear [5].

Our data and results for *Capitella* suggest that there are actual differences between larval and postlarval developmental programs. The question arises: which mode of segmentation, larval or postlarval, is more ancestral? Several authors [12,27] believe that larval segmentation (that is, the simultaneous metamerization of the somatic plate that creates larval segments) is a basic type, while sequential formation of postlarval segments from the GZ is an evolutionary novel segmentation type. Anderson, on the other hand, believes that postlarval segmentation is a primitive type [14,27].

During *A. virens* nectochaete development, only the ‘ancient’ *Hox* genes are expressed. Indeed, the minimal *Hox* gene complement of Urbilateria, the last common ancestor of bilateral animals, consisted of at least seven *Hox* genes. It contained five anterior genes (PG1-5), at least one gene from a central group (PG6/8), and at least one posterior gene (PG9+) [19]. This is exactly the set of *Hox* paralogs that is expressed during development of *A. virens* larva [4]. At the onset of postlarval growth, the genes of the central group, namely *Hox7*, *Lox4*, and *Lox2*, are activated. This may support the notion that postlarval segmentation is an evolutionary novelty. However, there is a certain contradiction with data for larval *Hox* gene expression in *Capitella*. All *CapI-Hox* genes are expressed during larval segment formation, including *CapI-Hox7*, *CapI-Lox4*, and *CapI-Lox2.*

The existing evidence is not sufficient to draw final conclusions about larval and postlarval developmental programs in the polychaetes. This emphasizes the need for *Hox* gene expression studies in other polychaete families, since this will probably elucidate the evolution of postlarval morphogenesis in this class.

### Possible *Hox* gene function in *A. virens* postlarval development

The nereid *A. virens* produces morphologically similar segments for almost its whole life. According to our data, most of *A. virens Hox* genes are expressed in the segment ectoderm and in the neural system of each postlarval segment. *Hox* gene transcripts are distributed as complex gene-specific gradients. As the worm grows, the position of each segment along the body AP axis changes, and this is accompanied by a change in *Hox* gene expression profile (Figure 11). We assume that the system of *Hox* gene transcriptional gradients is necessary not for specification of segment morphology, but to create and maintain the positional information for each segment. Within the constantly growing polychaete body, this regulatory system allows one to assign each metamere a unique ‘*Hox* code’, which gradually changes as the segment moves with respect to the terminal structures, that is, the head and pygidium (Figure 11). In this case, the ‘*Hox* code’ serves for positional, rather than morphological, specification.

Establishing and maintaining of positional information may be necessary for regeneration. Nereididae can regenerate posterior body parts. After a part of the body is lost, it is necessary to change the scale of the positional coordinates quickly in accordance with the new boundaries of the body. Since genes in the *Hox* cluster are often coordinated by common regulatory elements and a common feedback system, one can expect that the expression pattern of the whole complex would be easily reorganized during positional failure.

Planarian worms (Turbellaria; Platyhelminthes) also have gradient *Hox* gene expression. In planarian *Dugesia japonica*, *Plox4-Dj* (PG5; *Hox5*), *Plox5-Dj* (PG6-8; *Lox5*), and *Dj-Abd-Ba* (PG9+, Post2) transcripts are distributed as gradients along the AP axis of the adult worm [28,29]. The key aspect is that in this case *Hox* gene expression does not correspond to any morphological structures, has no clear boundaries and is proportional to the length of the worm body. It was also experimentally shown that *Dj-Abd-Ba* gene quickly restores expression pattern in head and tail regenerates, long before the new head and tail are formed, and this pattern is consistent with the new planarian body proportions [29].

Persistent 5' *HoxC* gene expression was surprisingly discovered in the spinal cord of an adult newt *Pleurodeles waltl*[30]. RT-PCR data showed that *PwHoxc13*, *PwHoxc12*, and *PwHoxc10* expression is upregulated during tail regeneration. The authors of this study suggest that *Hox* genes in an adult newt act as carriers for the positional memory necessary to achieve effective regeneration [30].

It is noteworthy that in newts and polychaetes the definitive *Hox* gene expression is associated with the neural system. Expression patterns of various *Hox* genes in the *A. virens* VNC ganglia are somewhat different (Figure 3). *Hox* gene function in *A. virens* is probably associated with specification of particular neuron types within VNC ganglia. Studies of another polychaete, *Platynereis dumerilii*, support this hypothesis. There is evidence that *Pdu-Hox* genes are expressed in different parts of the neuromeres [31]. Moreover, in the leech *Helobdella* expression patterns of different Нох genes in the ganglia are also different [24]. Colinear *Hox* gene expression was found during nervous system development in arthropods, vertebrates, ascidians, and annelids [5,32,33]. In some animals, the nervous system is the only structure that shows spatial and temporal colinearity of *Hox* genes expression [3,24,34]. Probably, this role in nervous system regionalization is the most conserved function of the *Hox* cluster.

### *Hox* genes cooptions in *A. virens*

Some *Alitta Hox* genes are likely to take part in patterning of particular structures rather than working in an orchestrated way together with other genes of the cluster. For example, *Nvi-Hox1* and *Nvi-Hox2* genes are expressed in the pharynx and at the esophagus-midgut boundary. Interestingly, their orthologs in other studied polychaetes, such as *Capitella* and *Chaetopterus*, are also expressed in the pharynx [5,7]. In addition, *Hox* orthologs in deuterostomes are involved in establishing the posterior boundary of pharyngeal entoderm [35,36]. This cooption is likely to date back to the early stages of evolution, probably to the origins of all Eubilateria.

*Hox1* orthologs work in peristomial and pygidial cirri in *A. virens* and *P. dumerilii*. Since *Hox1* expression is found in the notopodia and neuropodia of these polychaetes, one can assume that it is expressed in cirri as in serially homologous structures and thus is not purely a cooption. In general, most *Nvi-Hox* genes are expressed in parapodia, and this calls for a separate study. It is noteworthy, however, that this expression changes in accordance with general gradient pattern in the neural system and segment ectoderm of the worm.

The aforementioned *Nvi-Post1* gene was identified in parapodial chaetal sacs not only in *Alitta* and *Platynereis*, but even in *Capitella*[5,20], which is located far from Nereididae in the phylogenetic tree [37,38]. This suggests that this gene fell out of the common regionalization program early on.

In contrast to other Lophotrochozoa, *Hox* genes in *Alitta* have a rather limited range of cooptions. For example, *Nvi-Hox7* functions only in the neural system and in the segment ectoderm of postlarval segments, while its *Capitella* ortholog *CapI-Antp* has additional expression domains in the brain and pharynx [5]. In *P. dumerilii*, the *Lox2* gene works in the coelomic epithelium; this sets it apart from other *Pdu-Hox* genes and the *Nvi-Lox2* gene, which exhibits strong expression in ectodermal tissues [31].

In general, studied *Hox* gene cooptions in different species within the Lophotrochozoa clade support an idea that these genes are easily involved in new morphogenetic programs. They often play a role in the morphogenesis of structures, specific for large taxa within this animal group. For example, they pattern the shell gland in gastropods and the brachial crown in cephalopods [3,39].

### *Nvi-Hox* genes antisense transcripts

Our results represent the first discovery of *Hox* gene antisense transcripts in an animal from the Lophotrochozoa clade. These are long antisense RNA, complementary to the sense transcripts.

*Hox* cluster regulation by noncoding RNA was reported for mammals and arthropods [40,41]. Over 200 noncoding RNAs of different sizes and directions are transcribed from four human *Hox* clusters [40]. Our finding suggests that this is a universal regulatory mechanism in bilateral animals.

*The Nvi-antiHox5* transcript is expressed in a pattern almost complementary to the sense transcript (Figure 8). This resembles *anti-Ubx* expression in centipedes [41,42]. *Anti-Ubx* is functional in embryogenesis and exhibits a complex expression pattern that is mutually exclusive with the sense transcript. There have been no functional studies so far, but this early and specific nature of expression suggests a possible regulatory role of *anti-Ubx*.

The *Nvi-antiHox7* anterior expression boundary lies ahead of *Nvi-Hox7* (Figure 9). Both transcripts are detected in VNC ganglia and in the segment ectoderm, but their expression patterns are different within the same ganglia (Figure 9C,F). The mutually exclusive territories of sense and antisense transcript distribution suggest that one may be controlled by another.

So far, we have identified antisense transcripts for most *A. virens Hox* genes. Some of them are cloned (data are being prepared for publication). It should be noted that *Hox* genes long antisense RNAs in polychaetes are located in the cytoplasm. We do not know their function or biogenesis, but existing examples allow us to suppose that antisense RNAs take part in epigenetic tuning of the *Hox* cluster. They can participate in transcriptional and translational repression of their targets, protein-encoding RNAs. *Nvi-antiHox5* and *Nvi-antiHox7* expression patterns suggest that they may be controlling the anterior (*Nvi-Hox7*) or posterior (*Nvi-Hox5*) expression boundaries of their sense counterparts. The worm is constantly growing, and its *Hox* genes change levels of expression in a coordinated manner in accordance with the body proportions. In this situation, turning the genes on and off quickly at a translational level may be very advantageous.

## Conclusions

The data on expression patterns of *A. virens* and *Capitella sp.I Hox* genes support the idea of different morphogenetic programs underlying larval and postlarval development of the polychaetes. The larval program seems to be more conservative among the polychaetes. The principles of *Hox* gene expression during larval segmentation are similar to those during the body plan formation of other bilaterians. The postlarval programs display more differences between species and seem to be more evolutionary flexible.

The gradient expression of the *Nvi-Hox* genes in the postlarval period may be responsible for establishing and maintaining the positional values along the AP axis of multi-segmented, constantly growing polychaete body. The role of *A. virens Hox* cluster in positional specification can be studied in regeneration experiments.

The system of long antisense RNAs is a possible clue to the question of ‘sliding’ *Hox* gene expression boundaries in growing worms. This has not yet been described in existing model systems (planarian, mouse, drosophila), and we aim to investigate this phenomenon thoroughly.

## Abbreviations

AP: Anterior-posterior; asRNA: Antisense RNA; bp: Base pair; DTT: Dithiothreitol; GZ: Growth zone; RT-PCR: Reverse transcriptase polymerase chain reaction; VNC: Ventral nerve cord; WMISH: Whole-mount *in-situ* hybridization.

## Competing interests

The authors declare that they have no competing interests.

## Authors’ contributions

NIB, ELN, and MAK performed the experiments and data analysis. AYN participated in material collection, maintenance of the worm culture and editing the manuscript. ELN, NIB, and MAK conceived the study and drafted the manuscript. All authors have read and approved the final manuscript.

## Supplementary Material

Additional file 1 **Negative control with no probe.** All these worms were processed in the same WMISH. (**A,B,C**) The worms with different numbers of segments.**(D,E,F)** The posterior ends of 25 to 35 segment worms in higher magnification. The anterior end is to the right on all panels. All views are ventral. (A) The small worms do not display any background. (B-E) Weak background is detected in the gut (arrowheads), epithelial glands (arrows), parapodia, pygidial glands (red arrows) and at the ventral surface of the heads of 15 to 35 segment animals. (F) Some worms display strong background in the parapodia and pygidial glands. We have never seen any background in the nervous system or mesoderm. WMISH, whole-mount *in-situ* hybridization. Click here for file

Additional file 2 **Some details of *Nvi-Hox1 *expression. (A)***Nvi-Hox1* expression in the postlarval worm (overview). Images on panels **(B)** and **(C)** indicate the different expression patterns of *Nvi-Hox1* in the anterior (C) and posterior (B) ganglia of the VNC. **(D)***Nvi-Hox1* expression in the peristomial cirri. **(E)***Nvi-Hox1* expression in the pharynx and foregut-midgut boundary. VNC, ventral nerve cord. Click here for file
