# Supplementary material for: Hox gene expression during postlarval development of the polychaete Alitta virens
Source: EvoDevo. 2013 May 1;4:13. doi: 10.1186/2041-9139-4-13 (PMC3734159; doi:10.1186/2041-9139-4-13)

Negative control with no probe. All these worms were processed in the same WMISH. (A) (B) (C) The worms with different number of segments. (D) (E) (F) The posterior ends of 25–35 segment worms in higher magnification. The anterior end is to the right on all panels. All views are ventral. (A) The small worms do not display any background. (B)–(E) Weak background is detected in the gut (arrowheads), epithelial glands (arrows), parapodia, pygidial glands (red arrows) and at the ventral surface of the heads of 15–35 segment animals. (F) Some worms display strong background in the parapodia and pygidial glands. We have never seen any background in the nervous system or mesoderm.

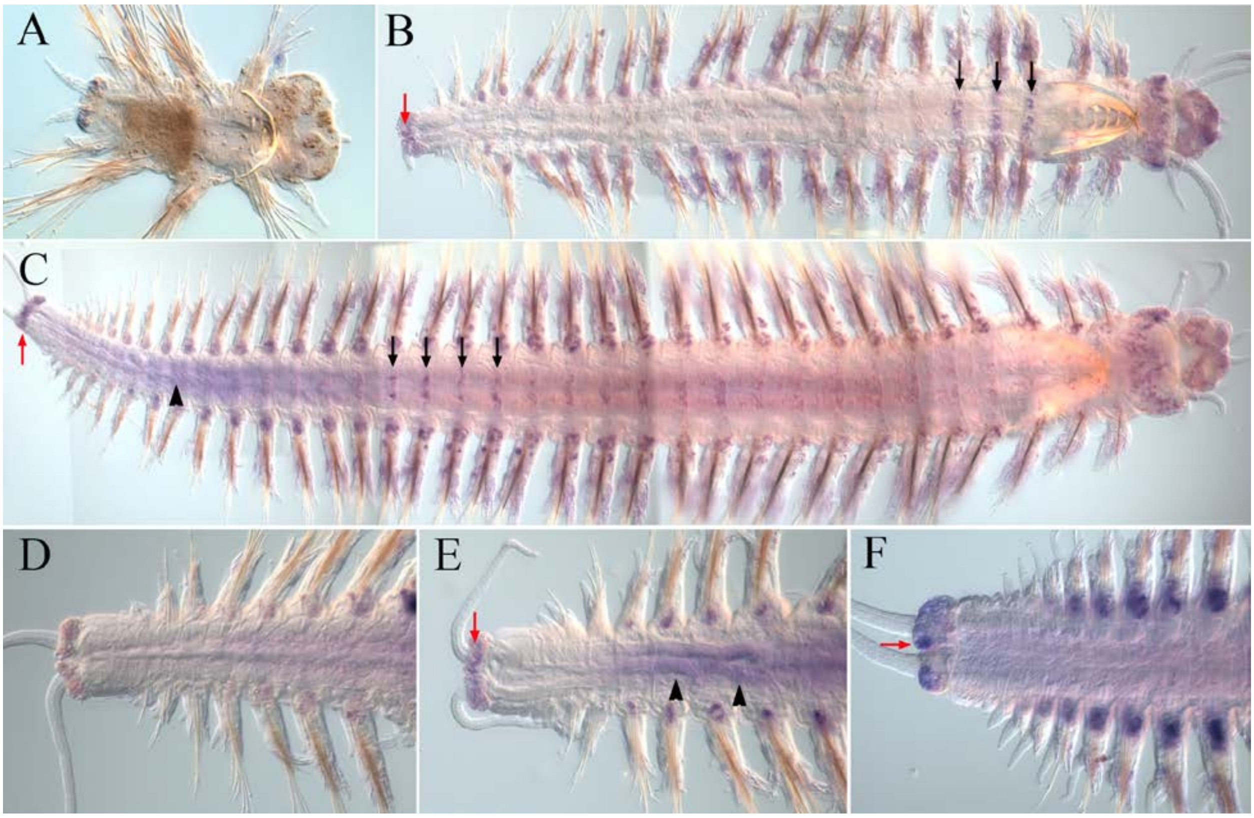

Supplement: Additional file 1 — Negative control with no probe. All these worms were processed in the same WMISH. (A,B,C) The worms with different numbers of segments.(D,E,F) The posterior ends of 25 to 35 segment worms in higher magnification. The anterior end is to the right on all panels. All views are ventral. (A) The small worms do not display any background. (B-E) Weak background is detected in the gut (arrowheads), epithelial glands (arrows), parapodia, pygidial glands (red arrows) and at the ventral surface of the heads of 15 to 35 segment animals. (F) Some worms display strong background in the parapodia and pygidial glands. We have never seen any background in the nervous system or mesoderm. WMISH, whole-mount in-situ hybridization. [file 2041-9139-4-13-S1.pdf]
