# Supplementary material for: Hox gene expression during postlarval development of the polychaete Alitta virens
Source: EvoDevo. 2013 May 1;4:13. doi: 10.1186/2041-9139-4-13 (PMC3734159; doi:10.1186/2041-9139-4-13)

Some details of *Nvi-Hox1* expression. (A) *Nvi-Hox1* expression in postlarval worm (overview). Images on panels (B) and (C) indicate the different expression patterns of *Nvi-Hox1* in the anterior (C) and posterior (B) ganglia of the VNC. (D) *Nvi-Hox1* expression in the peristomial cirri. (E) *Nvi-Hox1* expression in the pharynx and foregut-midgut boundary.

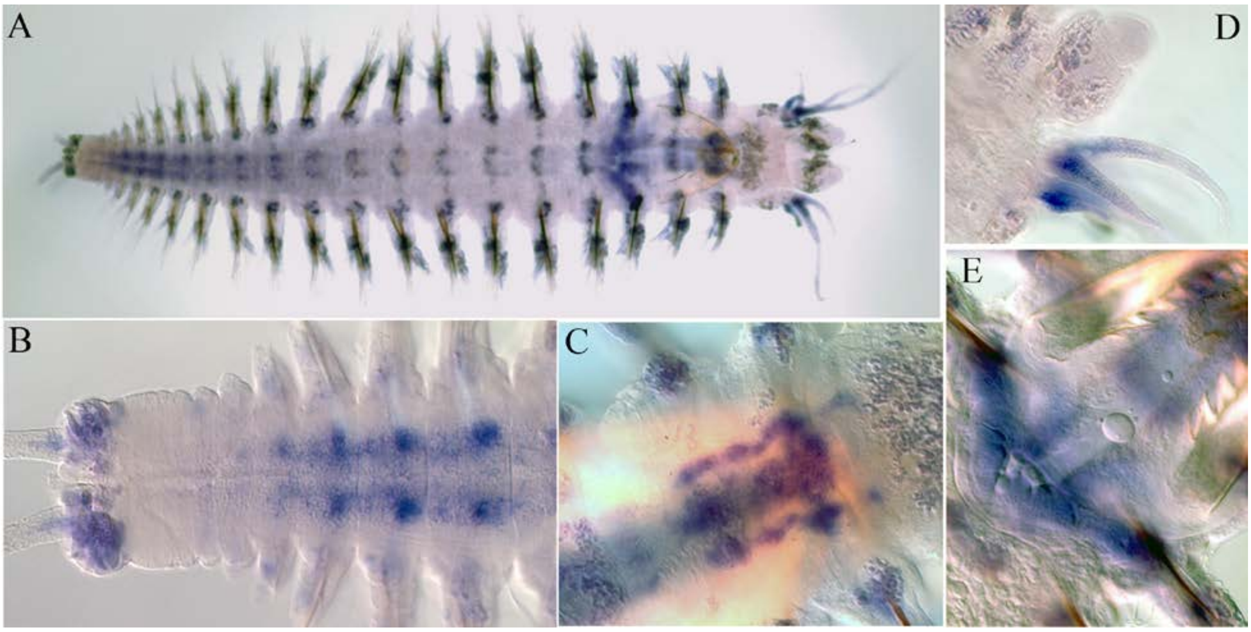

Supplement: Additional file 2 — Some details of Nvi-Hox1 expression. (A)Nvi-Hox1 expression in the postlarval worm (overview). Images on panels (B) and (C) indicate the different expression patterns of Nvi-Hox1 in the anterior (C) and posterior (B) ganglia of the VNC. (D)Nvi-Hox1 expression in the peristomial cirri. (E)Nvi-Hox1 expression in the pharynx and foregut-midgut boundary. VNC, ventral nerve cord. [file 2041-9139-4-13-S2.pdf]
